# QoS Measurement of Workflow-Based Web Service Compositions Using Colored Petri Net

**DOI:** 10.1155/2014/847930

**Published:** 2014-07-03

**Authors:** Hossein Nematzadeh, Homayun Motameni, Radziah Mohamad, Zahra Nematzadeh

**Affiliations:** ^1^Department of Computer Engineering, Islamic Azad University Sari Branch, Sari, Iran; ^2^Faculty of Computing, Universiti Teknologi Malaysia, 81310 Skudai, Johor, Malaysia

## Abstract

Workflow-based web service compositions (WB-WSCs) is one of the main composition categories in service oriented architecture (SOA). Eflow, polymorphic process model (PPM), and business process execution language (BPEL) are the main techniques of the category of WB-WSCs. Due to maturity of web services, measuring the quality of composite web services being developed by different techniques becomes one of the most important challenges in today's web environments. Business should try to provide good quality regarding the customers' requirements to a composed web service. Thus, quality of service (QoS) which refers to nonfunctional parameters is important to be measured since the quality degree of a certain web service composition could be achieved. This paper tried to find a deterministic analytical method for dependability and performance measurement using Colored Petri net (CPN) with explicit routing constructs and application of theory of probability. A computer tool called WSET was also developed for modeling and supporting QoS measurement through simulation.

## 1. Introduction

Composition of web services could be categorized in four major groups. One of these major groups is called workflow-based web service composition (WB-WSCs) in which most of the specification of QoS is neglected except performance and reliability [1]. WB-WSC includes eflow [2, 3], polymorphic process model (PPM) [4, 5], and BPEL [6–8]. Quality of service (QoS) which refers to nonfunctional parameters is important to be measured since the customers' requirements to a composed web service should be achieved. To do so, Colored Petri net (CPN) was extended and enhanced for further deterministic dependability and performance measurement. Even for BPEL, the background researches reveal that most of the QoS measurement methods were on performance and reliability [9, 10]. Studying the literature also reveals that three main specifications of Petri net and its extensions make them capable in modeling and evaluation of WB-WSCs; Petri nets have graphical notation; Petri nets' states could be shown explicitly, and there are many available techniques to evaluate Petri nets [11–16]. Therefore, explicit CPN was selected to evaluate WB-WSCs in this research. The explicitly of CPN is in using split/join transitions in case of conflict or concurrency. In order to measure the QoS firstly explicit CPN was defined and enhanced to include new routing constructs called PICK split/join. Then the components of WB-WSCs were mapped to explicit CPN using a given transformation table. Finally, deterministic QoS measurement was done in terms of availability, security, maintainability, reliability, dependability, and performance for WB-WSCs. The QoS measurement was done both analytically and experimentally using simulation. Firstly, the analytical formulas were developed based on theory of probabilities (independent and dependent probabilities) and geometric distribution; then, a case tool called WSET was also developed to apply simulation and find the experimental results. The experimental results supported and complied with the developed analytical formulas.

The organization of the remaining parts of the paper is as follows: in Section 2, the definition of CPN was provided and followed by enhancement in CPN routing construct. Then, a transformation table from WB-WSCs to CPN with explicit routing constructs was given. Next, Section 3 explained how dependability and performance can be calculated deterministically and analytically using CPN with explicit routing constructs. Then, Section 4 introduced WSET for simulation and proposed illustrative examples. Finally, the paper concluded with related discussions and future work on Section 5.

## 2. Transforming WB-WSCs to CPN

In this paper CPN was defined explicitly for the purpose of workflow modeling in web service compositions as a *N* = (*P*, *T*, *F*, *W*, *C*, *M*_0_, *S*, *τ*), where

- *P*  is a set of places. Places are shown using circles inside CPN which are responsible for collecting tokens;
- the set of transitions *T* is divided into two subsets: *T*_*i*_ and *T*_*t*_ defining the set of immediate and timed transitions, respectively. Immediate transitions fire immediately once they are enabled whereas timed transitions fire after a random enabling time in [*α*, *β*] in which *α* is the minimum and *β* is the maximum time assigned to each transitions. Formally *T*_*i*_ ∪ *T*_*t*_ ∈ *T*;
- *F* is a set of arcs known as a flow relation which are shown using arrows. *F*⊆(*P* × *T*)∪(*T* × *P*), that is, any two transitions either *T*_*t*_ or *T*_*i*_, cannot be linked directly to each other. They need a place between;
- *W* : *F* → *N*, *N* = 1,2, 3… is a set of arc weights, which assigns to each arc *f* ∈ *F*. In this research, the weights of all arcs were *N* = 1 denoting one token is consumed from a place by a transition or alternatively, one token is produced by a transition and put into each place;
- *C* = {*C*_1_, *C*_2_, *C*_3_,…, *C*_*k*_} is the finite set of color. The CPN in this research contains distinguished colored tokens which could be identified by their* id*s. Each* id* is a nonnegative integer. Arcs and transitions can carry and fire *C*;
- *M*_0_ : *P* → 0 is an initial marking, where for each place *p* ∈ *P*, there are 0 ∈ *N* tokens. There is a source transition (*T*_0_) which is responsible to fire new tokens periodically with distinguished* id*s into the CPN;
- *S* : 1 → *N*, *N* = 1,2, 3… assigns an integer number to any timed transition which specifies the size of that transition. Any transition could be considered as an array of transitions in this definition. The concept of size helps to model the concept of web service instances. At any time two different users can approach one web service simultaneously. This, for example, could be achieved if a transition has a size of two;
- *τ* → [*α*, *β*] assigns each timed transition a deterministic period of time. This gives a delay to each timed transition which helps to calculate the overall performance of a token when it reaches final place(s).

The guard in timed transitions is always true (timed transitions fire any nonnegative integer* id* as colored tokens) and the expression attached to the arcs was defined in a way that any arcs could carry any nonnegative integer* id* as colored tokens. All of the CPN places can have data values of integers.

### 2.1. Enhancement in CPN Routing Constructs

**Figure 1:**
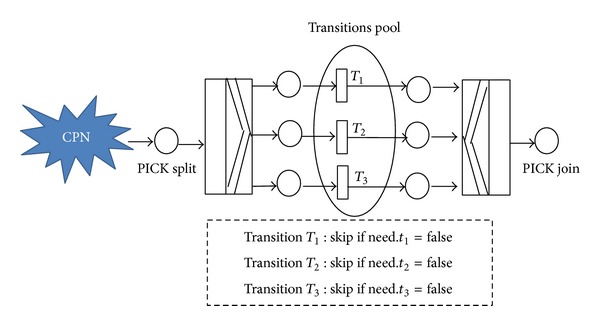
The Schematic of PICK split/join with three transitions in generic pool.

Generally there are two ways to model systems using Petri nets: implicit modeling and explicit modeling. If there exists a situation (triggers or external application,…) in the system that leads making decision occurs as late as possible, then implicit modelling will be used. When making decision to select a route in CPN that is clear then explicit modeling would be selected. For explicit modeling researchers usually use AND split/join transitions for concurrency and OR split/join transitions for conflict (exclusive OR). The new building block called PICK split/join was proposed in this research to model the concept of inclusive OR explicitly. PICK split selects one or more routes from many routes in contrast with traditional OR split which selects exactly one route from many routes. Application of inclusive OR usually occurs while having several services in the form of checkboxes as in generic node in eflow. The firing rule of PICK split/join was given in the following. The schematic block also was given in Figure 1.

- A PICK split is said to be enabled if the input place *p* of split transition contains at least one token.
- Based on the routing rules inside PICK split, outcoming arcs should be fired. The choice is based on workflow attributes; it is a deterministic choice.

PICK split can skip any of three transitions (*T*_1_, *T*_2_, *T*_3_) in transitions' pool, using a boolean variable like need. The term “transitions' pool” refers to number of transitions that potentially could be selected.

### 2.2. Mapping from WB-WSCs to CPN

**Figure 2:**
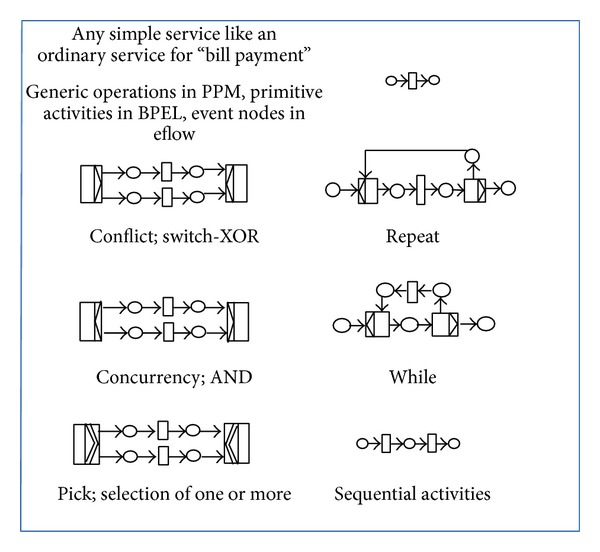
Explicit transformation of WB-WSCs to CPN routing constructs.

A global explicit transformation model from WB-WSCs (eflow, PPM, and BPEL) to CPN was given in this section and depicted in Figure 2. Basic service node in eflow, primitive activities of BPEL, and activities and subprocesses of PPM were transformed to a transition in CPN. It is important to mention that BPEL does not support the concept of inclusive OR. Using the transformation model of Figure 2, the final CPN model would be free choice and well structured. By the term free choice it was meant that there is not any confusion in CPN and well structuredness refers to using AND join transitions for AND split, OR join transitions for OR split, and PICK join transitions for PICK split in synchronization [17]. Figure 2 clearly showed how primitive services and structured services can be transformed into the related CPN model. Structured services are inclusive OR, exclusive OR, concurrency, loop (repeat/while), or sequential activities. Generally parallel routing of web services (executing all routes) is shown with AND split transition and its synchronization is shown using AND join transition. Exclusive conditional routing/XOR (executing exactly one route from many) is shown with OR split transition and its synchronization is shown using OR join transition. Conditional routing/OR (executing at least one route from many) is shown with PICK split and its synchronization is shown with PICK join.

## 3. Dependability Measurement

**Figure 3:**
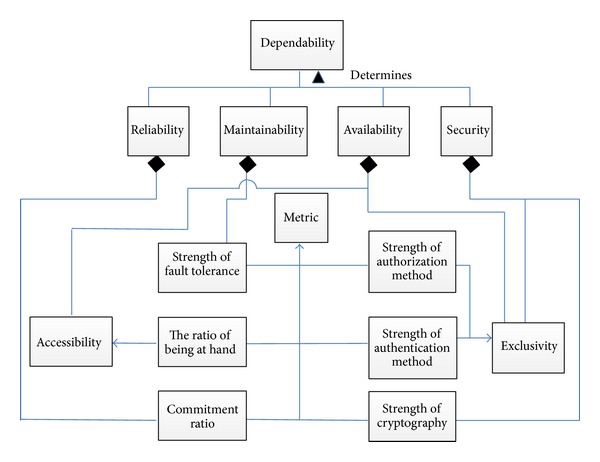
Dependability conceptual model.

Reliability is the probability of the WB-WSC system to carry out its functional requirements failure-free for a specified period of time and under stated conditions. Reliability can be assessed by calculating the commitment ratio of a service by finding the percentage a certain service could commit in a specified period of time. Security is the probability of the WB-WSC system to provide authenticated users or services with access to the services under certain authorization. Authentication and authorization referred to exclusivity. Not only exclusivity is a matter of concern in security but also the method which is used for encrypting data is a key metric in security of a service in a WB-WSC. It is possible to specify methods for measuring exclusivity and data encryption method through the supporting party [18]. Maintainability is the probability of the WB-WSC to recover itself in a specified time (*t*) in case of failure [19]. The fault tolerance methods comprising error detection and error discovery techniques used in the services of WB-WSC are a key factor in determining maintainability. Availability in WB-WSC means both exclusivity and accessibility. Only legitimate users and parties should access the service in WB-WSC and the service that is accessed should be usable. Accessibility ensures accessing usable services. Usability was shown by the term “at hand” in Figure 3. One way to assess the accessibility is to calculate the percentage of time the service becomes deliverable within the determined time [20]. Dependability is in the top of the hierarchy. The more reliable, secure, maintainable, and available the WB-WSC is, the more dependable it is [21]. Since reliability, security, maintainability, and availability have direct impact on dependability, the average of the four already defined quality attributes was defined as dependability in WB-WSCs. Supporting parties can manage and monitor the correctness of the metrics in Figure 3 that the service provider claims. The existence of a supporting party guarantees the level of service for service users from service providers. This could be done by updating WSLA service definition and service obligation. Based on the metrics and the related discussions above and the definition of quality attributes in the literature [22, 23], the overall dependability conceptual model based on the key features of each parameter was given in Figure 3 represented in UML 2.0.

### 3.1. Analytical Calculation Process

Transitions in CPN could either success or fail to fire. In this view transitions led to two outcomes: 1 for success and 0 for failure. In an uncertain view, a probability of success rate could be assigned to each transition through testing. Assuming 0.98 as a success rate of the transition *C* means that transition *C* would be able to fire successfully 98 times in 100 times. So, the failure rate would be 0.02 whereas 0.98 + 0.02 = 1. Through this strategy the success rate of each of the metrics in Figure 3 can be calculated via a third supporting party. The probability of success and failure was determined in (1) as follow:

(1) P(success)=the  number  of  success100,P(failure)=the  number  of  failure100.

In which *P*(success) + *P*(failure) = 1. Knowing the metrics, the quality parameters in Figure 3 can be calculated. The calculated metrics assigned to CPN transitions were independent. Regarding reliability, surely the commitment ratio of transition* A* does not depend on the commitment ratio of transition* B*. However, the composition of transitions may affect the quality calculation as follows.

**Figure 4:**
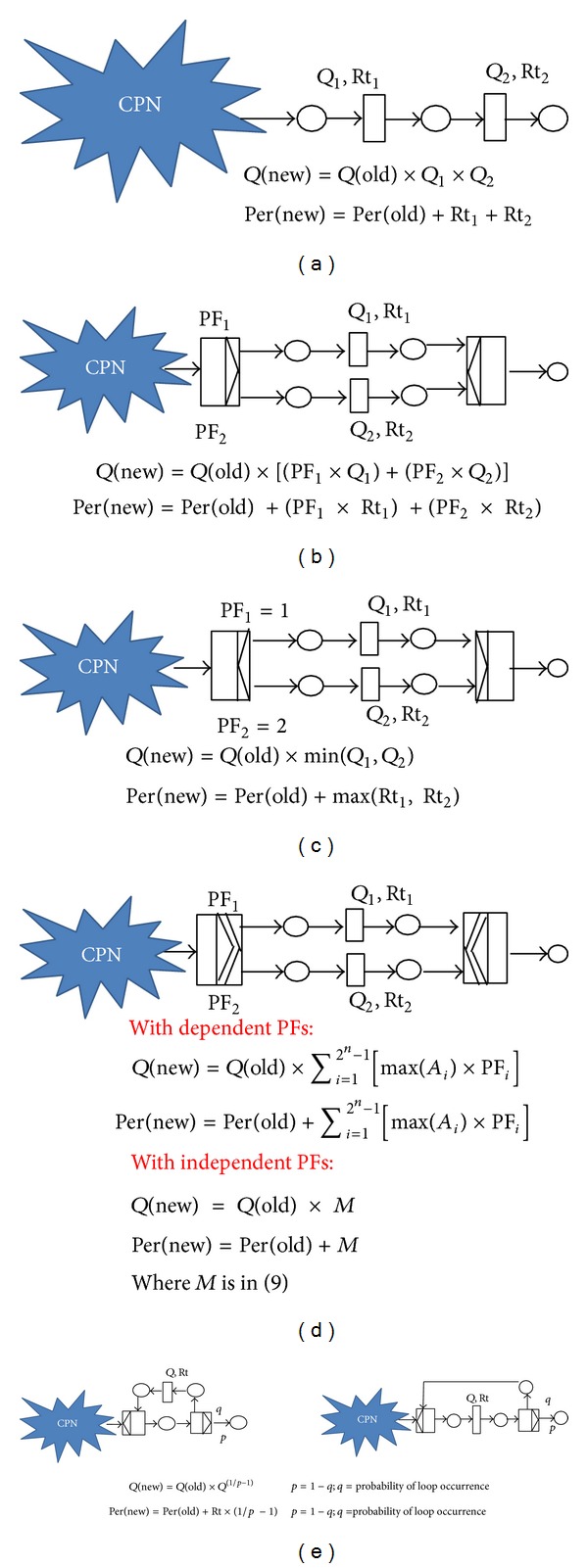
Quality calculation process on CPN routing constructs using theory of probability.

*(A) Sequential Transitions*. Assuming the CPN in Figure 4(a) has two sequential transitions with the quality metrics of *Q*_1_ and *Q*_2_, respectively, the new quality value (*Q*(new) at the output place of transition with *Q*_2_) was calculated through the multiplication of *Q*_1_ and *Q*_2_ by *Q*(old) in (2) where *Q*(old) is the calculated quality inside CPN (*Q*(old) is increased with successive multiplication in CPN). This is because *Q*_1_ does not depend on *Q*_2_; therefore, based on the independent theory of probability, the overall quality would be the multiplication of *Q*_1_ by *Q*_2_. In addition, the performance of CPN using sequential transitions in Figure 4(a) can be calculated as in (3) where Rt_1_ and Rt_2_ are the average response times of current transitions. It is important to mention that the initial value of *Q*(old) and *Per*⁡(old) (at the beginning of CPN) was set to one and zero, respectively. Always at the end of calculation of each quality calculation equation, *Q*(new) and *Per*⁡(new) should be assigned to *Q*(old) and *Per*⁡(old), respectively, until reaching the end place of CPN. Consider

(2) Q(new)=Q(old)×Q1×Q2,

(3) Per⁡(new)=Per⁡(old)+Rt1+Rt2.

*(B) Conflict (Exclusive OR)*.  Figure 4(b) illustrated a part of CPN with OR split/join blocks of transition. Generally, the quality value inside OR split/join block can be calculated through summation of multiplication of probability of firing (PF) of each outcoming arc in OR split by its respective quality value as shown in (4) and Figure 4(b). Consider

(4) Q(new)=Q(old)×[(PF1×Q1)+(PF2×Q2)].

Split/join transitions are nonblocking so they do not have any impact in the overall performance of the CPN. If a maximum and minimum time for the timed transitions could be identified and also the probability of firing of each outcoming arc of split transitions could be specified, the performance would be calculated deterministically using the average time and probability of firing. Assuming the average response time for transition with *Q*_1_ and *Q*_2_ is Rt_1_ = 6 and Rt_2_ = 8, respectively, in Figure 4(b); the token inside CPN (in the input place of OR split) has the average response time of *n* = 12; OR split has the probability of firings of PF_1_ = 0.6 and PF_2_ = 0.4  (PF_1_ + PF_2_ = 1). The quality value, which here is the performance in the output place of OR join, will be calculated as 12 + (0.6 × 6)+(0.4 × 8) = 18.8 using (5) in which *Per*⁡(new) means new performance and *Per*⁡(old) means old performance. Consider

(5) Per⁡(new)=Per⁡(old)+(PF1×Rt1)+(PF2×Rt2).

*(C) Concurrency*. Regarding AND split transitions, PFs for all outcoming arcs are one; therefore, the quality value inside AND split/join transitions is the minimum of quality values inside AND split/join block. Likewise, *Q*(new) was calculated through multiplication of min (*Q*_1_, *Q*_2_) by *Q*(old) as shown in (6) and Figure 4(c). Since the quality value was intended to be calculated in the worst case, the minimum of (*Q*_1_, *Q*_2_) were chosen. Consider

(6) Q(new)=Q(old)×min⁡(Q1,Q2).

However, in performance calculation of AND split/join, the maximum time of transitions between AND split and AND join is considered because performance was intended to be calculated in the worst case. Therefore the overall performance of Figure 4(c), with Rt_1_ = 6 and Rt_2_ = 8, would be calculated as 12 + *Max*⁡((1 × 6), (1 × 8)) = 20, using (7) as follows:

(7) Per⁡(new)=Per⁡(old)+Max⁡(Rt1,Rt2).

*(D) Selection of One or More (Inclusive OR).* Two approaches were identified in prediction of the behavior of PICK split/join blocks: (1) using dependent PFs for PICK split and (2) using independent PFs for PICK split.  Figure 4(d) showed the average quality value calculation in case of having PICK split/join blocks of transition with dependent PFs. In Figure 4(d), *A* is a set that has all subsets of set *C* except *Ø*, so it had 2^*n*^ − 1 members. *A*_*i*_ is the *i*th member of *A*, PF_*i*_ is the *i*th member of PF, PF is the set of probability of firing for each member of *A*, and *C* is a set of all quality values between PICK split/join. Hence, in Figure 4(d), *A* = {(*Q*_1_), (*Q*_2_), (*Q*_1_, *Q*_2_)}, *C* = {*Q*_1_, *Q*_2_}, and assuming probability of firings of PF = {0.4,0.25,0.35} (PFs are dependent), then the quality value inside PICK split/join would be *Q* = (0.4 × *Q*_1_) + (0.25 × *Q*_2_) + (0.35 × min⁡(*Q*_1_, *Q*_2_)) accordingly. The minimum was used to calculate the quality between PICK split/join transitions in the worst case. Therefore the *Q*(new) is the summation of multiplication of *A*_*i*_ by its respective PF_*i*_ multiplied by *Q*(old) as shown in

(8) Q(new)=Q(old)×∑i=12n−1[min⁡(Ai)×PFi].

As a second way of calculation of the quality parameter inside PICK split/join, independent probability of firings could be assigned to each outcoming arc of PICK split. For example, in Figure 4(d), two probabilities of 75% and 60% could be assigned to PF_1_ and PF_2_, respectively (the sum of PF_1_ and PF_2_ is not necessarily 1). In implementation view, for each output arc of PICK split, a random number in [1,100] would be generated. If the random generated number is in the range of the probability of the path then that path will be selected. In this case in Figure 4(d), PICK split will not fire with the probability of 10% = (1 − 75%)×(1 − 60%). Thus, PICK split transition should refire to send out all existing tokens. Using independent probabilities, the average quality value inside PICK split/join block can be calculated as (9) where *n* is the number of transitions between PICK split/join. Consider

(9) M:The  probability  of  occurring  all  2n−1  cases1−The  probability  of  repeating  the  experiment,M:(PF1×(1−PF2)×Q1+PF2×(1−PF1)×Q2+ PF1×PF2×min⁡(Q1,Q2)) ×(1−[(1−PF1)×(1−PF2)])−1.

Since the quality value was intended to be calculated in the worst case, minimum of (*Q*_1_, *Q*_2_) was selected. In addition, the *Q*(new) calculation in Figure 4(d) changes as follows:

(10) Q(new)=Q(old)×M.

In case of using PICK split/join with independent PFs (assuming PF_1_ = 60%, PF_2_ = 50%) and with Rt_1_ = 6 and Rt_2_ = 8, the overall performance would also be calculated as 12 + (5.8/0.8) = 12 + 7.25 = 19.25 through (9) and (11) as follows:

(11) M:(PF1×(1−PF2)×Rt1+PF2×(1−PF1)×Rt2+ PF1×PF2×min⁡⁡(Rt1,Rt2))  ×(1−[(1−PF1)×(1−PF2)])−1,Per⁡(new)=Per⁡(old)+M.

The total performance inside PICK split join block also can be calculated using dependent PFs as in (12) where *A* is a set that has all subsets of set *C* except *Ø*, so it had 2^*n*^ − 1 members. *A*_*i*_ is the *i*th member of *A*, PF_*i*_ is the *i*th member of PF, PF is the set of probability of firing for each member of set *A*, and *C* is a set of all response time values between PICK split/join. Consider

(12) Per⁡(new)=Per⁡(old)×∑i=12n−1[max⁡(Ai)×PFi].

*(E) Loop.* For quality calculation inside loop, geometric distribution in theory of probability was used. In geometric distribution, parameter *p* is a geometric distribution parameter where the range of *p* is 0 < *p* ≤ 1. Now if the probability in which OR split fires token inside loop is known as *q*, then *p* could be simply calculated as

(13) p=1−q.

Using the probability mass function in (14), the exact number of (*k* − 1) times OR split transition fires a certain token inside loop can be calculated as follows:

(14) (1−p)k−1×p k:1,2,3….

However, since the deterministic calculation was intended in this research, the mean geometric distribution was used as in (15) to find the average time a certain token can be fired inside loop through OR split. Consider

(15) 1p.

Nevertheless, assuming *q* = 0, then *p* = 100%, the average time the token can be fired inside loop through OR split is calculated as 1/100% = 1. When *q* = 0, then 1/*p* should be zero. Thus, (15) should change to (16) to alleviate this problem. Consider

(16) 1p−1.

Generally the deterministic quality value of loop (repeat/while) can be calculated using (17) where *Q* is the amount of quality value inside loop. Consider

(17) Q(1/p−1).

Thus, the overall quality value of a token after passing a loop block diagram can be calculated as (18) and also was illustrated in Figure 4(e). Consider

(18) Q(new)=Q(old)×Q(1/p−1).

In which *p* = 1 − *q* and *q* = probability of loop occurrence. Obviously, the quality of each transition was calculated based on the metrics for each parameter. For example, if the intended quality is security then *Q* = strength of authorization method × strength of authentication method × strength of cryptography. In implementation of loop, a random number is generated in OR split block. If the random number is in the range of *q* (probability of loop occurrence), the token will be fired inside loop through OR split transition. In addition, the performance of CPN using loop in Figure 4(e) can be calculated as in (19) where Rt in (19) is the average response time of loop. Consider

(19) Per⁡(new)=Per⁡(old)+Rt×(1p−1),p=1−q; q=probability  of  loop  occurrence.

It is important to mention that using dependent PFs, the characteristic of inclusive OR was changed to exclusive OR. This is not handy especially when the number of outcoming arcs of PICK split is increased. Assuming 5 members for set *C* yields 31 members for set *A* and PF. The advantage of considering independent PFs is easier calculation and simpler implementation. The implementation concept of PICK split using independent probabilities of firings was shown for given two outcoming arcs (PF_1_ = 0.75 and PF_2_ = 0.6) in Algorithm 1(a). Algorithm 1(a) was modified with more memory savings in Algorithm 1(b) and was generalized in Algorithm 1(c).

## 4. Simulation and Illustrative Examples

Web service evaluation tool, WSET, was developed by java programing to support explicit and deterministic QoS measurements of WB-WSCs experimentally. Using WSET the WB-WSC designer can first generate the intended eflow, PPM, and BPEL. Then, WSET converts the WB-WSC to CPN for intended QoS measurement, namely: performance, reliability, availability, security, maintainability, and dependability. Regarding the QoS measurement, based on the time of *T*_0_ (source transition) and final time (the entire time the CPN should be simulated) which is specified by the user, the CPN simulation is done by WSET. The public method Token () included attributes for QoS measurement which are initiated when the public method is called in each run of WSET. As the colored tokens pass the transitions of CPN the quality values will be computed accordingly and at the end of simulation the amount of each quality value in end places of CPN will be aggregated and averaged. The data structure that is mostly used in WSET was stack. All the places of CPN have the first in first out queue in order to keep the colored tokens. Source transition in CPN which is responsible to fire tokens inside the system also uses first in first out queue. In order to detect the routing constructs of CPN (split/join transitions), a first in last out queue is associated with a token.

### 4.1. ECS

**Figure 5:**
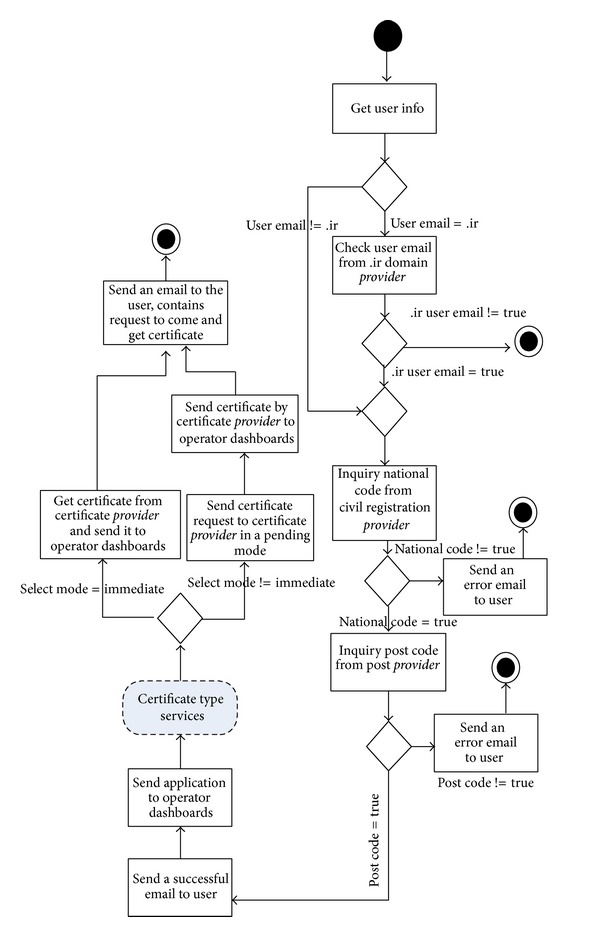
Eflow of ECS.

**Figure 6:**
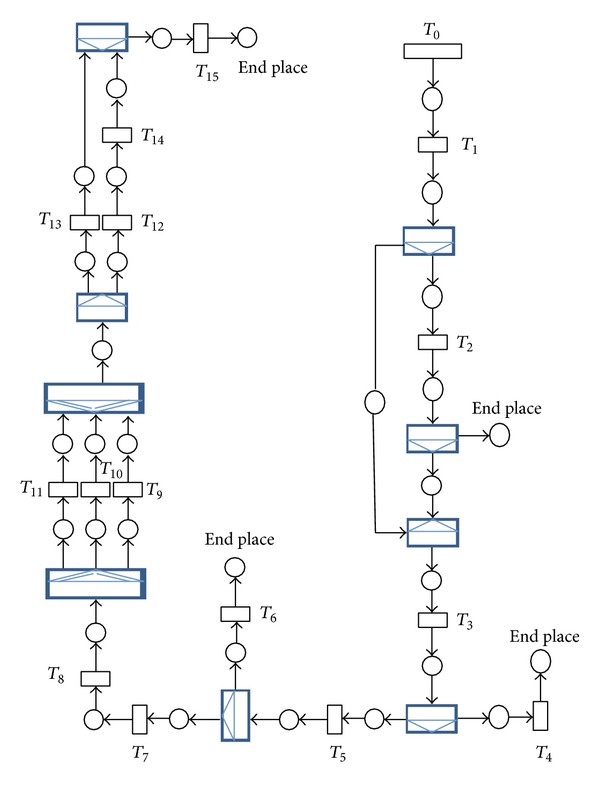
CPN of eflow of ECS.

Electronic certificate service, (ECS), was a sample project implemented in the center of issuing of electronic certificates in Iran ministry of commerce in which its eflow was illustrated in Figure 5 and the relevant CPN was given in Figure 6. The user commences preregistration process through entering personal information, national code, post code, and email. In case of success in (user info collection, user email domain verification, national code verification, and postcode verification), a verification email would be sent to the user in which the user would be asked to send back the necessary documents for issuing certificate. Then, the filled application would be sent to operator dashboards. The operator will do the definitive registration by selecting a certain certificate from the certificate pool (SSL, secure email, digital signature). Next, operator will select the definitive certificate issuance mode (immediate or pending). To notify the user regarding the certificate issuance, an email would be sent to the user contains a request which implies to come and receive the certificate.  Table 1 shows the result of WSET for measuring performance, reliability, and availability of the eflow; based on the given inputs PICK split/join was calculated based on the independent probabilities of outcoming arcs. According to Table 1 the minimum and maximum time for transition *T*_0_ is 10 and 30, respectively. This means that on the average each (10 + 30)/2 = 20 units of time each colored token can enter the CPN through *T*_0_. Using 20000 units of time for final time, the approximate number of colored tokens can enter the CPN can be calculated as 20000/20 = 1000. Final time refers to the simulation period. A web service is modeled by a transition however its instance was modeled by the concept of size which was assumed 10 for each timed transition. This means that any timed transition can service to 10 colored tokens simultaneously. In other words there will be no queues for 10 colored tokens in the input place of a transition with the size of 10.

**Figure 7:**
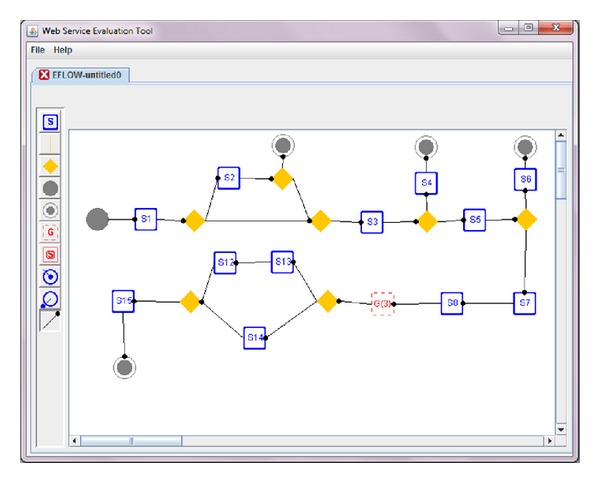
Generation of ECS using WSET.

**Figure 8:**
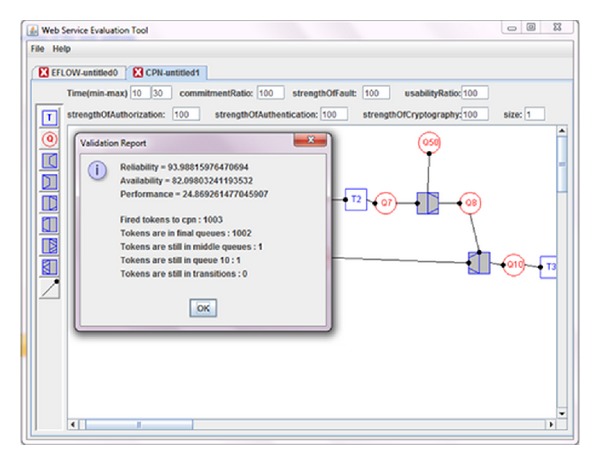
QoS measurement of ECS using WSET based on the given input.

The metrics in Table 1 (min time, max time, CR, SAM, SAM(2), and BH) can be calculated and managed by supporting parties. The responsibility of supporting parties is to guarantee the levels of service (quality of service) that the service provider provides for its customer. Regarding the calculation of commitment ratio one of the strategies that the supporting party can apply is to run the special service at least 100 times with different test cases to check the percentage the special service commits in a given period of time. That would be the commitment ratio for reliability calculation. The rest of the metrics can also be calculated using the same strategy. Also supporting third parties can propose these levels of service in web service level agreement, so by fetching the WSLA of each web service by service users this information could be achieved.  Figure 7 also shows how WSET generates ECS. Likewise, Figure 8 shows WSET QoS measurement results after transformation the initial ECS to the relative CPN using Figure 2.

As it is clear in Figure 8, WSET gives extra information besides the intended QoS. WSET also shows how many colored tokens have been fired to the CPN through *T*_0_ and how many of them could reach to the final queues based on the final time which was assumed to be 20000. With 20000 for final time, 1003 colored tokens have been fired to the CPN through the source transition *T*_0_ which had minimum time of 10 and maximum time of 30. Likewise, WSET could identify the number of tokens which have not reached to the final queues because of time limitation which was specified by final time. The experimental result in Figure 8 could also be calculated using analytical formulas in Section 3.1. In fact the nondeterministic behavior of CPN was predicted deterministically provided that the probability of firings and the metrics for intended quality attributes were given and the explicit view in CPN (using split/join transitions) was considered.

### 4.2. UTSP

**Figure 9:**
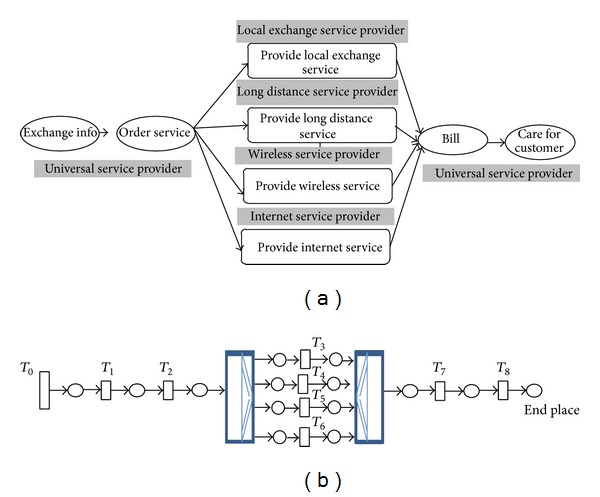
(a) PPM of UTSP and (b) CPN for PPM of UTSP.

Universal telecommunication service provisioning (UTSP) process is a famous PPM-based multienterprise process which was illustrated in Figure 9(a) and the CPN was shown in Figure 9(b). The top process starts when a customer requests a universal telecommunication services by providing the information via a web browser through exchanging information activity. For verification of information and creation of the corresponding record, order service activity is performed. Next, the top process continues with a combination of four subprocesses. After all selected subprocessess are completed an activity is performed to create a single bill. Finally, the care for customer activity informs the establishment of the requested universal service and verifies if it meets the customer needs.  Table 2 shows the result of WSET for measuring performance, maintainability and security of the entire PPM based on the given inputs. Final time was assumed 5000, size of transitions was assumed 10 and independent probability of firing was used for PICK split transition. Based on the minimum and maximum time of *T*_0_ 1000 tokens (5000/5) will be fired approximately through *T*_0_ to the CPN on the average.

### 4.3. DES

**Figure 10:**
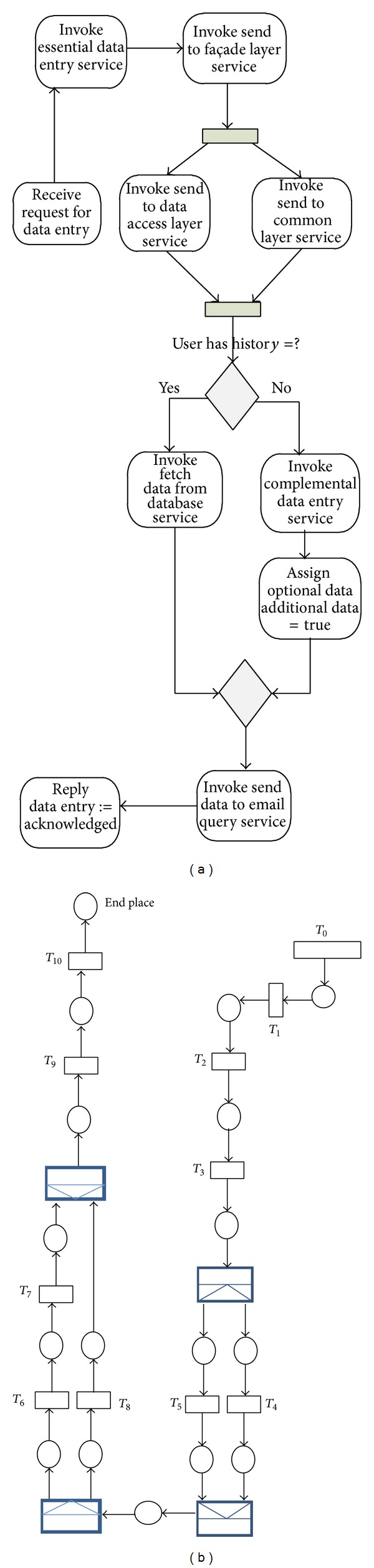
(a) BPEL of DES and (b) CPN for BPEL of DES.

Data entry service (DES) is an orchestration service that is used for monitoring entered data. Such a service can be used in any data collection process. The relevant BPEL of a possible DES was given in Figure 10(a) and its CPN was given in Figure 10(b). First, initial and essential data were inserted through invoking essential data entry service. Then, for retrieval from central database and to do some further analysis, the inserted data was sent to façade layer which is responsible for managing services and in parallel to common layer and data access layer. Then to find a possible history from the inserted data the fetch data from database service was invoked. In case of no history the complemental data entry service was invoked and the additional data were saved and also was assigned true using assign optional data. Finally a service would send all data to email query. Suppose that the BPEL designer/modeler wants to calculate the dependability, reliability, availability, security, maintainability and performance of the given BPEL based on the inputs in Table 3. Here, the final time was assumed 20000, size of transitions were assumed 10, and dependent probabilities of firing were used and adjusted for OR split outcoming arcs in which 30% of time fetch data from data base service was invoked.

## 5. Discussion

Generally what have been done in this research was QoS measurement of WB-WSCs. ECS, UTSP, and DES were selected for evaluation. Then evaluation was done with the help of WSET. WSET can be initiated with arbitrary number of colored tokens and at the end the average result regarding QoS of WB-WSCs complied with the deterministic formulas calculated in Section 3.1 analytically. QoS depends on the customer's view. Business should try to provide good quality regarding the customers' requirements to a composed web service. Through this research it was shown how this quality can be calculated numerically using the theory of probability including dependent probabilities, independent probabilities, and geometric distribution for a WB-WSC. Instead of using fuzzy terms like the dependability of the BPEL is good or the availability is satisfactory, now it can be said that the dependability is almost 90% and the availability is approximately 81%. In this way the terms good and satisfactory were defuzzified and even between two good results the better one can be chosen. It is clear that we are looking for “just enough” [24] level of quality in WB-WSCs as a software-based system. There is a balance between web services cost, schedule, and the expected level of quality. But at least we can predict the overall QoS in a web service composition to see if it meets the customer needs or not. Of course for having more qualified WB-WSCs, more qualified web services should be used which could increase the service cost and the delivery schedule. WSET supported the QoS measurement result experimentally and proved the analytical formulas. Using WSET, WB-WSC designer/modeler can also generate web service compositions with nested structured activities. It was also mentioned how supporting third parties can calculate the needed metrics and attach them to the WSLA of each web service. In this way each metric could also be found by fetching the related WSLA. The technique proposed in this research could be used only if the probability of firings in OR split/PICK split is known by the developer or web service composer. In fact this yields explicit modeling in CPN in which split transitions could be used. The reason three different cases were selected was that first, neither all the structured activities could not be shown in one example nor all the QoS was intended in all the cases.

Regarding WSET a questionnaire and the case tool were sent to the experts in the field. The questionnaire contained 6 questions regarding the functionality of WSET on its user-friendliness, WB-WSC generation and conversion to CPN, QoS measurement, usefulness, novelty of the tool, and originality of the tool. The experts were asked to give 1 to 4 to the questions which one resembled weak, two resembled satisfactory, three resembled good, and four resembled excellent. WSET received the average result of 3.31 from 4 in which the average result on user-friendliness was 3, WB-WSC generation and conversion to CPN received 3.5, QoS measurement received 3.5, usefulness received 2.8, originality received 3.8 and the novelty of the tool received 3.3. The related works in WB-WSCs testing and evaluation are not much on eflow and PPM. However, there are researches on BPEL testing. But the majority of Petri net based researches on evaluation of BPEL had limitations as stated in Table 4. One of the main limitations of the previous works was that eflow could not be validated based on quality parameters since eflow has the concept of generic node in which in CPN it could be mapped to PICK split/join which was introduced in this research. Through this research the following major achievements were obtained:

- enhancement of routing constructs in explicit CPN with PICK split/join transition,
- deterministic analytical QoS measurement of WB-WSCs,
- developing WSET as a case tool for modeling and experimentally measuring QoS of WB-WSCs.

Currently, there are four directions for future works:

- applying implicit methods in modeling and QoS measurement of WB-WSCs where decision making cannot be shown explicitly and split/join transitions cannot be used in the modeling,
- evaluation of other workflow-based systems such as UML activity diagram and BPMN using the analytical formulas and modeling methods of this research,
- congestion detection in WB-WSCs using the concept of size in CPN transitions,
- calculation of dependability using a nonlinear function.

## Figures and Tables

**Algorithm 1:**
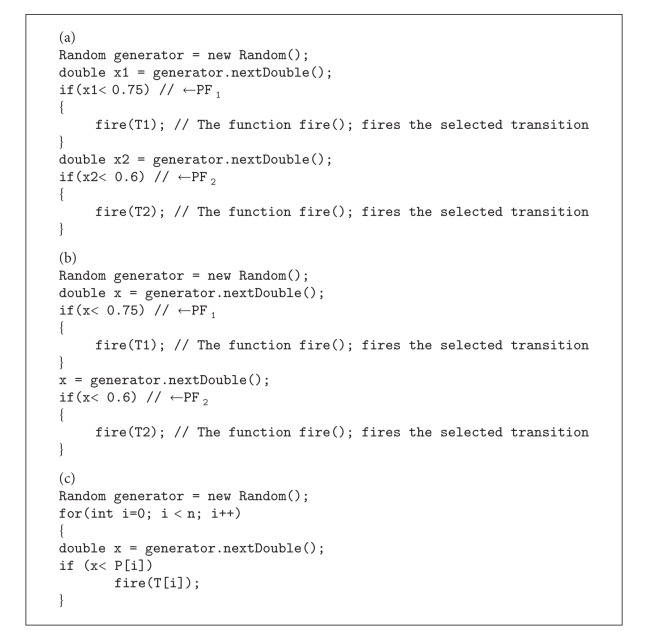
PICK split firing rules (a): for two outcoming arcs, (b): for two outcoming arcs and more memory saving, and (c): generalized for *n* outputs.

**Table 1:** Quality evaluation of eflow using WSET.

Transition	Minimum time	Maximum time	CR	SAM	SAM(2)	BH
*T*_0_	10	30	100	100	100	100
*T*_1_	2	5	99	100	100	98
*T*_2_	2	4	99	99	99	100
*T*_3_	3	4	99	98	99	99
*T*_4_	1	2	100	100	100	100
*T*_5_	3	4	99	98	99	99
*T*_6_	1	2	100	100	100	100
*T*_7_	2	3	100	100	100	99
*T*_8_	2	3	100	100	100	99
*T*_9_	3	4	99	100	100	99
*T*_10_	3	4	99	100	100	99
*T*_11_	2	4	99	100	100	99
*T*_12_	2	4	99	100	100	99
*T*_13_	3	5	99	99	99	99
*T*_14_	2	4	99	98	97	99
*T*_15_	2	3	99	100	100	98

PICK split probability of firings				30-50-30		
OR split probability of firings in order of appearance in Figure 6				(30-70), (5-95), (5-95), (5-95), (30-70)		
Size of transitions *T*_1_ to *T*_15_				10		
Final time				20000		
Reliability				93.98%		
Availability				82.09%		
Performance				24.86 (units of time)		

CR: commitment ratio, SAM: strength of authorization method, SAM(2): strength of authentication method, and BH: the ratio of being at hand (usability ratio).

**Table 2:** Quality evaluation of PPM using WSET.

Transition	Minimum Time	Maximum Time	Strength of fault tolerance	Strength of authorization method	Strength of authentication method	Strength of cryptography
*T*_0_	2	8	100	100	100	100
*T*_1_	4	8	99	99	98	100
*T*_2_	3	6	99	100	100	100
*T*_3_	12	16	98	99	99	99
*T*_4_	10	18	97	99	99	99
*T*_5_	8	13	97	98	98	99
*T*_6_	8	15	99	98	99	99
*T*_7_	3	6	100	100	100	100
*T*_8_	2	4	99	100	100	100

PICK split probability of firings				30-10-20-80		
Size of transitions *T*_1_ to *T*_8_				10		
Final time				5000		
Maintainability				95.50%		
Security				94.35%		
Performance				30.53 (units of time)		

**Table 3:** Quality evaluation of BPEL using WSET.

Transitions	*T*_0_	*T*_1_	*T*_2_	*T*_3_	*T*_4_	*T*_5_	*T*_6_	*T*_7_	*T*_8_	*T*_9_	*T*_10_
Minimum Time	10	2	6	5	6	5	6	8	2	5	2
Maximum Time	30	4	10	10	12	14	10	14	4	8	4
Commitment ratio	100	100	99	99	98	99	97	99	99	99	100
Strength of fault tolerance	100	100	99	100	100	100	100	99	100	99	100
Strength of authorization method	100	100	99	99	99	99	99	98	100	99	100
Strength of authentication method	100	100	98	99	99	99	98	98	100	99	100
Strength of cryptography	100	100	99	99	99	99	98	98	100	99	100
The ratio of being at hand (Usability ratio)	100	99	99	99	99	99	99	99	99	99	99

OR split probability of firings						30-70					
Size of transitions *T*_1_ to *T*_10_						10					
Final time						20000					
Maintainability						97.34					
Reliability						92.88					
Security						88.01					
Availability						81.48					
Dependability						89.93					
Performance						50.72 (units of time)					

**Table 4:** Comparison of literature methods and proposed method.

Method	QoS Measurement	Scope in WB-WSCs	Type of PN	Case Tool
Proposed method	YES dependability, reliability, maintainability, security, availability, and performance	Eflow, PPM, BPEL	Explicit CPN	YES
Zhong and Qi, 2006 [8]	Only performance and reliability	BPEL	Implicit SPN	NO
Dong et al., 2009 [6]	Only performance	BPEL	Implicit SPN	NO
Chen et al., 2009 [9]	Only reliability	BPEL	Implicit PN	NO
Song et al., 2009 [7]	Only performance	BPEL	Implicit TPN	NO
